# Draft genome of a vancomycin-resistant *Enterococcus faecium* recovered from a bloodstream infection

**DOI:** 10.1128/mra.00518-25

**Published:** 2025-06-12

**Authors:** Ying Zeng, Donglai Dai, Yu Feng

**Affiliations:** 1 Department of Pharmacy, The People’s Hospital of Neijiang Dongxing District, Neijiang, Sichuan, China; 2 Department of Clinical Laboratory, The People’s Hospital of Neijiang Dongxing District, Neijiang, Sichuan, China; 3 Laboratory of Pathogen Research, West China Hospital, Sichuan University 34753, Chengdu, Sichuan, China; Queens College Department of Biology, Queens, New York, USA

**Keywords:** vancomycin-resistance, blood, *Enterococcus faecium*

## Abstract

We report the draft genome sequence of a vancomycin-resistant *Enterococcus faecium*, isolated from a patient with bloodstream infection. The strain was classified as sequence type 80 and carries the *vanA*-type operon. The final assembled genome assembly comprises 243 contigs totaling 2,915,356 bp, with a GC content of 37.66%.

## ANNOUNCEMENT

Vancomycin-resistant *Enterococcus faecium* (VREfm) represents a critical global public health threat, characterized by limited therapeutic options (1). Recent genomic surveillance studies revealed that the emergent sequence type (ST) 80 has globally replaced the previously dominant ST17 lineage, gaining colonization advantages through bacteriocin-mediated inhibition of competing gut enterococci (2). Therapeutic management is especially challenging since VREfm not only resist vancomycin but frequently harbor additional resistance determinants, rendering many first-line antibiotics ineffective (3). These highlight the clinical and epidemiological significance of VREfm and emphasize the necessity of detailed genomic characterization.

In February 2025, a blood specimen from a patient with bacteremia at The People’s Hospital of Neijiang Dongxing District was inoculated onto Luria–Bertani (LB) agar and incubated aerobically at 37°C for 18 hours. The recovered bacteria were streaked for isolation, and a single, well-separated colony was then transferred into LB broth and incubated under same conditions. This study has been approved by the Ethical Committee of The People’s Hospital of Neijiang Dongxing District, with informed consent being waived. Genomic DNA was subsequently extracted using the QIAamp DNA Blood Mini Kit (Qiagen, Hilden, Germany). A 150 bp paired-end library with an average insert size of 300 bp was prepared using the NEBNext Ultra II DNA Library Prep Kit (New England Biolabs, Ipswich, MA, USA) and sequenced on the Illumina NovaSeq 6000 platform (Illumina, San Diego, CA, USA). The sequencing run yielded 5,612,122 read pairs totaling 1.68 Gbp, achieving 577 × genome coverage. Raw reads were quality-trimmed using Trimmomatic v0.39 (4), applying a minimum leading and trailing base quality of Q10. The filtered reads were then assembled with SPAdes v4.0.0 (5) in isolate mode, resulting in a genome consisting of 243 contigs totaling 2,915,356 bp, with a GC content of 37.66% and an N*50* of 33,322 bp. Species identification was confirmed with FastANI v1.34 (6), demonstrating an average nucleotide identity of 99.19% to *E. faecium* type strain DSM 20477^T^ (accession no. CP118955.1). Antimicrobial resistances (AMR) were identified using AMRFinderPlus v4.0.22 (7), and the ST was assigned using the web service from the Center for Genomic Epidemiology (8). Virulence genes were screened using BLASTn against the core *Enterococcus* virulence data set from the VFDB (9). The genome annotation was performed by the NCBI Prokaryotic Genome Annotation Pipeline v6.10 (10). Unless stated otherwise, all programs were run with default parameters.

The strain was assigned to *E. faecium* ST80. *In silico* analyses predicted multiple AMR genes (Table 1) and three adherence-associated virulence factors: the collagen-binding gene *ecbA*, the collagen adhesin precursor gene *acm*, and the cell-wall-anchored adhesin gene *sgrA*. In accordance with CLSI breakpoints (11), antimicrobial susceptibility testing conducted using the Vitek II automated microbiology system (bioMérieux, Marcy-l’Étoile, France) confirmed its resistance to ampicillin (MIC ≥ 32 mg/L), ciprofloxacin (MIC ≥ 8 mg/L), tetracycline (MIC ≥ 16 mg/L), and vancomycin (MIC ≥ 32 mg/L), while the strain remained susceptible to linezolid (MIC = 2 mg/L) and tigecycline (MIC ≤ 0.12 mg/L).

**TABLE 1:** Antimicrobial resistance genes identified in *Enterococcus faecium* strain 005010

Class/Gene	Product
Amikacin/Kanamycin	
*aph(3')-IIIa*	Aminoglycoside O-phosphotransferase APH(3')-IIIa
Aminoglycoside	
*aac(6')-I*	Aminoglycoside 6′-N-acetyltransferase
Azithromycin/Erythromycin/Streptogramin B/Tylosin	
*msr(C*)	ABC-F type ribosomal protection protein Msr(C)
Clindamycin/Erythromycin/Streptogramin B	
*erm(B*)	23S rRNA (adenine(2058)-N (6))-methyltransferase Erm(B)
Streptomycin	
*ant (6)-Ia*	Aminoglycoside nucleotidyltransferase ANT (6)-Ia
*ant (6)-Ia*	Aminoglycoside nucleotidyltransferase ANT (6)-Ia
Streptothricin	
*sat4*	Streptothricin N-acetyltransferase Sat4
Tetracycline	
*tet(S*)	Tetracycline resistance ribosomal protection protein Tet(S)
Vancomycin	
*vanA*	D-alanine--(R)-lactate ligase VanA
*vanH-A*	D-lactate dehydrogenase VanH-A
*vanR-A*	Vancomycin resistance response regulator transcription factor VanR-A
*vanS-A*	Vancomycin resistance histidine kinase VanS-A
*vanX-A*	D-Ala-D-Ala dipeptidase VanX-A
*vanY-A*	D-Ala-D-Ala carboxypeptidase VanY-A
*vanZ-A*	Glycopeptide resistance protein VanZ-A

## Data Availability

The draft genome assembly of strain 005010 has been deposited in GenBank under the accession number JBNOXI000000000. The raw sequencing reads have been submitted to the Sequence Read Archive under accession number SRR33417414.
